# Long-term efficacy with deep brain stimulation of the globus pallidus internus in cervical dystonia: a retrospective monocentric study

**DOI:** 10.1186/s42466-022-00214-8

**Published:** 2022-10-03

**Authors:** Clemens Jacksch, Kirsten E. Zeuner, Ann-Kristin Helmers, Karsten Witt, Günther Deuschl, Steffen Paschen

**Affiliations:** 1 Department of Neurology, Christian Albrechts-University of Kiel, Arnold-Heller-Str. 3, 24105 Kiel, Germany; 2 Department of Neurosurgery, Christian Albrechts-University of Kiel, Kiel, Germany; 3 Department of Neurology and Research Center Neurosensory Science, Carl Von Ossietzky University Oldenburg, Oldenburg, Germany

**Keywords:** Idiopathic cervical dystonia, Deep brain stimulation, Long-term effectiveness

## Abstract

**Background:**

Cervical dystonia (CD) is characterized by involuntary contractions of the cervical muscles. Data on long-term effectiveness of deep brain stimulation (DBS) are rare. The aim of this study was to evaluate the longitudinal ten years treatment efficacy of DBS in the globus pallidus internus (GPI).

**Methods:**

A retrospective single-center data analysis was performed on patients with idiopathic CD, who were treated with GPI DBS for at least 10 years. TWSTR severity score and individual sub-items were compared between pre and post DBS surgery (n = 15) over time.

**Results:**

There was a significant and persistent positive effect regarding the severity of TWSTRS between the conditions immediately before and 1, 5, and 10 years after establishment of GPI DBS (mean difference: 6.6–7 ± 1.6). Patients with increasing CD complexity showed a poorer response to established treatment forms, such as injection of botulinum toxin and were thus DBS candidates. Especially a predominant torticollis was significantly improved by DBS.

**Conclusion:**

GPI DBS is an effective procedure especially in severely affected patients with a positive 10-year outcome. It should be considered in more complex CD-forms or predominant torticollis.

## Background

Cervical dystonia (CD), as the most common form of adult-onset focal dystonia, is characterized by involuntary contractions of the cervical muscles which lead to abnormous movements or postures [1]. The prevalence varies between a range of 28–183 cases/million with about twice as many women affected as men [2]. Although most forms are idiopathic, „CD mimics“ have to be excluded, e.g. vascular, musculoskeletal, infectious or traumatic causes [1, 3]. Clinically CD presents with combinations of head and/or neck rotation, tilt or shift. Tremor may occur as a head or an arm tremor [4]. The severity of CD is objectively assessed either with the Toronto Western Spasmodic Torticollis Rating Scale (TWSTRS) or the Tsui-score [5–8].

First line therapy is the injection of botulinum toxin (BoNT). Additional treatment possibilities include oral drugs (e.g., anticholinergics). More severely affected patients or those who do not respond sufficiently to the standard treatment may benefit from deep brain stimulation (DBS) of the globus pallidus internus (GPI) [9, 10]. The improvement of dystonic symptoms has a direct influence on health-related quality of life, but 10% of the GPI DBS patients show no response [11].

Long-term data on the effect of GPI stimulation on CD over a longer period are rare. However, single studies were able to provide evidence for a good long-term efficacy with a mean follow-up time of 7.8 and 11.5 years (and an improvement from up to 47.6–54.1% in the TWSTRS severity score). Yet, they are difficult to compare, due to low patient numbers and inconsistent use of scores and endpoints [12, 13]. Other studies provided similar results, but their follow-up periods were either shorter (between 2 and 5 years) [14–17] or the number of patients rather small (n = 4) [18]. A shorter duration of illness [14] and a more phasic than tonic pattern [12] turned out to be possible positive prognostic factors. As a result, some authors suggested neck mobility might be a possible prognostic factor for the preoperative evaluation of suitable patients [19].

In this study, we analyzed the long-term efficacy of GPI-DBS over 10 years and attempted to characterize the CD patients who would particularly benefit from this treatment modality.

## Methods

### Collection of clinical data

A retrospective single-center data analysis was performed on patients with idiopathic CD, who were treated with GPI-DBS for at least 10 years. We retrieved our data from our electronic patient files and our movement disorder data base. All patients, who were treated in our Movement disorder clinics between 1999 and 2011 were included. Diagnosis was made by two movement disorder specialists in our clinic. Stimulation devices used in patients, who received GPI DBS included models from Medtronic (Activa® PC and RC pulse generator).

The following inclusion and exclusion criteria were applied:

### Inclusion criteria

Patients with a confirmed diagnosis of idiopathic cervical dystonia who had a history of GPI DBS for at least 10 years. Patients with dopamine-sensitive cervical dystonia (termed Segawa syndrome) were not considered for analysis. None of our patients used for the analysis were found to have any of the previously known genetic alterations for a possible monogenic inherited dystonia. Also, no patients with a "dystonia plus syndrome" were included, who had another dominant disease feature in addition to dystonia (for example, myoclonus or parkinsonism). However, genetic testing was performed in this group of patients in only a very small proportion, as standard panel testing had not been established at the time of surgery.

All patients must have received regular clinical follow-up visits (at least once a year) with collection of the TWSTRS severity score over this period. If multiple TWSTRS were collected in a time range, the value of the clinical visit closest to the 1, 5, and 10-year treatment period was used. The accepted range to assign the score to a specific time-point was ± 3 months in 1-year visit, ± 6 months in 5-years visit and ± 1 year in 10-years visit.

### Exclusion criteria

- stimulation targets other than GPI or stimulation of a second target point (n = 2)
- incomplete follow up data (n = 44)
- parallel treatment with DBS and BoNT (n = 2).

From the 63 CD patients who underwent GPI DBS surgery between 1999 and 2011, 15 patients with CD and fulfilled inclusion criteria remained.

To avoid potential confounders, patients with parallel botulinum toxin treatment were excluded. The reasons why these patients received BoNT in parallel were a poor response to the GPI DBS (poor responder) and concomitant pain. There are various reasons why patients did not show up for their scheduled clinical visits. As a reference center, we carry out many DBS surgeries every year, but the follow-up care of a considerable number of patients takes place close to their place of residence in other neurological clinics, which offer the follow-up care but not the surgery itself. In addition, those patients for whom the TWSTRS was not collected during the clinical visits or for whom the recordings could not be retrospectively reconstructed also dropped out of the analysis.

**Fig. 1:**
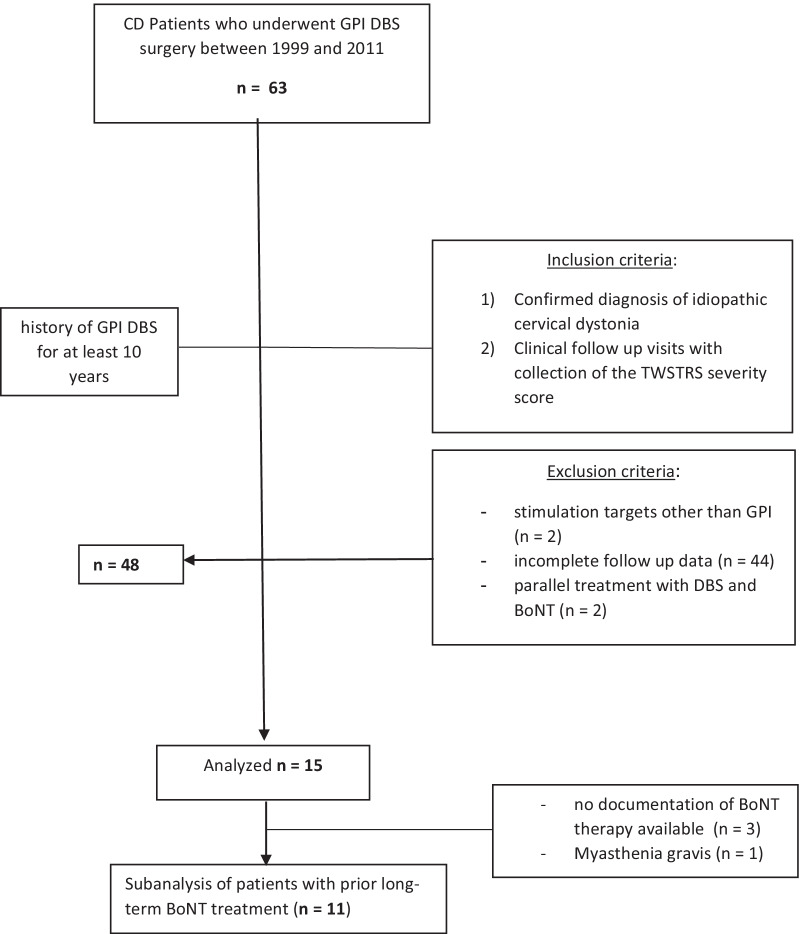
Consort patient flow of the study population

The Consort patient flow in Fig. 1 gives an overview of the study population.

For objective monitoring of disease severity, we used the TWSTRS severity score, which is our standard assessment during regular clinical visits.

The TWSTRS, as our validated rating scale, includes subitems for motor function, disability, and pain. The motor part, as we used in this study, has 0–35 points, the disability part 0 to 30 points and the pain part 0–20 points, with higher values indicating more severe symptoms. The TWSTRS-severity estimates the clinical CD presentation (torticollis, laterocollis, anterocollis, retrocollis, duration, shoulder elevation, range of voluntary rotational head movement, sensory trick).

The TWSTRS severity score recorded during clinical visits was compared over time between the different time points. An improvement of at least 25% on the TWSTRS was considered as a clinically relevant response [11].

Furthermore, we conducted a sub-analysis to compare sub-items of the TWSTRS severity score before and after DBS surgery in a group of eleven patient, who had been treated with BoNT long term in our hospital prior to surgery.

### Statistical analysis

The data were analyzed with the freely available program "JASP" (version 0.14, University of Amsterdam, Amsterdam, The Netherlands). In addition to the descriptive data analysis, repeated measures ANOVA with accompanying Greenhouse–Geisser correction and post-hoc testing at the 5% level were implemented to determine the long-term effect. P values < 0.05 were considered statistically significant. To examine a significant difference regarding the individual TWSTRS sub-items, paired t- tests were performed. The literature search with pubmed used the keywords "idiopathic cervical dystonia", "deep brain stimulation", "botulinum toxin" and "long-term effectiveness".

## Results

### Descriptive

The clinical baseline characteristics of the study population are shown in Table 1. The patients treated with GPI DBS had a mean age of 61.5 years, a slight dominance of the female sex (53.3%) and a mean disease duration of 26.6 years. Interestingly, both the amplitude (2.8/3.05 V), frequency (143.3/ 139.3 Hz) and pulse width (93/ 92 µs) used remained relatively constant over the years.

**Table 1:** Clinical features of CD patients and long-term treatment with GPI DBS

No.	Sex/Age	CD length (years)	DBS surgery in xx year of disease	BoNT pre-treated in our clinic	1°/2°. non-response to BoNT	Mode	Amplitude year 1/10 after DBS surgery (V)	Frequency year 1/10 after DBS surgery (Hz)	Pulse width year 1/10 after DBS surgery (µs)	ADM
1	Female/60	19	13	Yes	2°	DM	3.0/3.0	180/125	90/90	None
2	Male/61	17	4	Yes	2°	M	2.5/2.2	130/180	90/90	None
3	Male/54	22	9	Yes	2°	M	2.7/3.9	130/130	120/90	BA
4	Female/58	9	1	Yes	1°	IL	2.8/3.8	130/125	90/90	None
5	Male/70	50	38	Yes	2°	M	2.6/2.7	130/130	90/90	BA
6	Female/56	11	6	No	–	M	4.0/4.0	130/130	120/90	None
7	Male/55	21	6	No	–	M	3.45/3.3	180/180	105/120	None
8	Female/67	41	33	Yes	2°	DM	2.25/2.35	130/130	60/60	None
9	Male/68	26	20	Yes	2°	M	3.25/3.05	180/180	120/120	None
10	Male/70	50	36	Yes	2°	M	2.5/2.6	130/130	90/90	None
11	Female/44	24	10	Yes	2°	M	3.25/3.25	180/130	90/120	None
12	Male/58	35	19	No	–	DM	2.0/1.4	130/130	60/60	None
13	Female/53	22	10	Yes	2°	M	2.5/2.7	130/130	90/90	None
14	Female/63	Unknown	Unknown	Yes	1°	M	1.8/3.8	130/130	60/60	None
15	Female/85	26	11	No	–	M	3.8/3.75	130/130	120/120	None
Mean ± SD	Female: 53.3% Male: 46.7% Age: 61.5 (44; 85)	26.6 (9; 50)	15.4 (1; 38)	Yes: 73.33% No: 26.67%	2°: 81.8% 1°: 18.2%	M: 73.3% DM: 20% IL: 6.7%	2.8/3.05	143.3/139.3	93/92	BA: 2

ADM: additional anti dystonic medication; DM: double monopolar; M: monopolar; BA: beta agonist; IL: interleaving

Possible stimulation-induced side effects most frequently included the occurrence of dysarthria (5/15) and parkinsonoid (2/15). In addition, two cases required surgical revision due to cable traction and one infection in the area of the pulse generator necessitating its replacement.

Patients who never had a satisfactory result with BoNT prior to surgery were considered as primary non-responders (defined as a reduction of the severity score of 2 points or less after the first or second BoNT injection). Secondary non-responders included those patients, who had obtained at least 2 successful injections of BoNT (assuming a reduction of the severity score by at least 3 points and/ or adequate weakness of the injected muscles) [20].

### Long-term efficacy

**Fig. 2:**
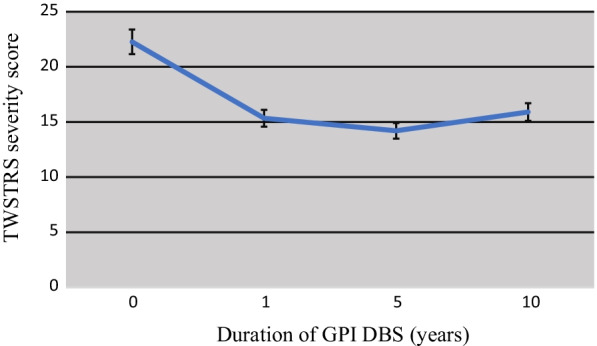
Course of TWSTRS severity score over 10 years in patients treated with GPI DBS

Descriptive data analysis already suggested a higher pre-DBS TWSTRS severity score compared with 1, 5, and 10 years after surgery. Repeated measures ANOVA reported a significant decline (p < 0.001) after Bonferroni correction that remained stable over 1, 5 and 10 years (mean difference 6.6–7.0 points ± 1.6 when comparing pre-DBS TWSTRS with assessments 1, 5 and 10 years of ongoing GPI DBS). The course of TWSTRS severity score in the DBS group over the 10-year period is illustrated in Fig. 2.

Of the 15 patients studied, one patient was a DBS "non-responder" (TWSTRS before and 10 years after surgery 24 and 25 points, respectively). This corresponds to a rate of approximately 6.7%, 93.3% of patients responded well for 10 years. A valid response rate cannot be given based on the available case numbers. In larger randomized studies, the response rate was more than 80% after 5 years [20].

Interestingly, the effect of GPI DBS coincides relatively well with the benefit from botulinum toxin injection known from the current literature (ΔGPI DBS: 6.6–7.0 ± 1.6; ΔBoNT: 6.4–6.6 [21]) as measured by the TWSTRS severity score.

### Clinical course in patients changing from BoNT to GPI DBS

Of the 15 patients in the GPI DBS group, eleven received BoNT therapy in our outpatient clinic prior to DBS surgery. From the remaining 4 patients, no documentation of BoNT treatment was available for 3 patients (referral from an external clinic), one patient suffered from myasthenia gravis and was thus not eligible for BoNT. The mean BoNT-treatment time was 10 years (min. 1 year; max. 20 years). All of them showed increasing complexity of their dystonic symptoms during the preoperative period. Especially these patients with increasing complexity and a predominant torticollis made the decision in favor for DBS. Table 2 shows exemplary the disease courses in 4 of the 11 patients. This should illustrate how CD can become more complex over years. More complex dystonia was defined as follows: (i) an increase in motor score as measured with the TWSTR; (ii) additional dystonic features (for example laterocollis/retrocollis/anterocollis in addition to torticollis; torticollis in addition to previously retrocollis or laterocollis etc.); (iii) The dystonic presentation increased in severity (for example torticollis started initially with 30 ^○^ and worsened to > 45^○^, or a laterocollis presented with 10°, and increased to 30^○^). In addition, higher complexity may also be related to poorer treatability of CD by BoNT, despite an initial usually good response.

**Table 2:** Examples of four disease courses during long-standing BoNT treatment and after DBS surgery

Patients characteristics (sex/age)	Initial finding	Development during long-term BoNT	Just before DBS surgery	Change after DBS surgery
w, 60	Dystonic „no–no“ tremor, phasic/myocloniform dystonic activity of the left shoulder; TWSTRS 14	Initially in 5-year course good response with only mild TC; TWSTRS 9	After approx. 10 years pronounced TC as well as RC and LC; TWSTRS 18	Tremor markedly improved, only subtle TC and LC, no RC described; TWSTRS 13
m, 68	Moderately severe dystonia with predominant AC (30°) and TC (30°)	Relatively stable course, new LC (pre BoNT 60°, post BoNT 30°), TC and AC at the same level as before; TWSTRS 10	In particular, increase in LC and pronounced shoulder elevation, TWSTRS 26 points. Severe neck pain	Improvement of all individual components, TWSTRS 18 points, favorable effect on pain
m, 56	Moderate TC (30°) and light LC (15°), also moderately severe shoulder elevation, TWSTRS 16 points	Mainly stable course, TWSTRS after 7 years 16 points	Higher complexity with increase in LC to 16°–35°, newly added AC with retrocaput, TWSTRS 20 points	Improvement with now still slight TC (20°) and LC (15°), TWSTRS 16 points
w, 61	Moderate cervical dystonia with TC (20°) and LC (20), TWSTRS 11 points	Deterioration of TC to 45°–67°	Further deterioration in TC to 68°–90°, TWSTRS 23 points	Continued moderate-severe TC (45°–67°), also slight lateral shift and problems crossing the midline, TWSTRS 18 points

TWSTRS: Toronto Western Spasmodic Torticollis Rating Scale; TC: Torticollis; RC: Retrocollis; LC: Laterocollis; AC: Anterocollis

In interpreting the different clinical courses, a dystonic (head) tremor could be suspected as a possible additional reason for the decision to GPI DBS, which is not captured in the TWSTRS.

7 of 15 patients had concomitant dystonic head tremor, which improved good to excellent in five patients. One patient had a subjective worsening after DBS surgery and one patient had a constant finding in regard to the tremor.

A sub-analysis of the DBS effect on the TWSTRS and its subitems of these 11 patients was conducted. Since BoNT is the standard treatment and in most cases initially applied to most of the patients, we intended to present the DBS effect in this subgroup. There was a significant and clinical meaningful decreasing in TWSTRS of 27.4% after one year of GPI DBS. Thus, this was comparable to the effect of the overall group.

We applied another paired t-test to compute the DBS-effect on the individual TWSTRS-subitems in this subgroup of patients. There was a statistically significant improvement of torticollis 12 months after surgery by an average of 1.1 points (56%; p = 0.021). In other words, an improvement of 1.1 points means a torticollis reduction of 22° according to the TWSTRS. The range of voluntary head motion showed a trend (p = 0.05) to an improvement of approximately 60% (mean difference 0.67 points). Further sub-items revealed no significant differences. The tendency, however, was in favor for an improvement in a pre-existing shoulder elevation from up top 45,6%, as well as an improvement in an anterocollis and retrocollis (-16.5%) (compare Table 3).

**Table 3:** Percentage change of the individual TWSTRS subitems 12 months after DBS surgery

TWSTRS subitems	Percentage change one year after DBS surgery (%)	p =
Torticollis	− 56	**0.021**
Laterocollis	± 0	1.00
Ante-/retrocollis	− 16.5	0.051
Lateral shift	+ 100	0.594
Shoulder elevation	− 45.6	0.141
Crossing midline	− 60	0.05

Significant results in bold at p < 0.05

TWSTRS: Toronto Western Spasmodic Torticollis Rating Scale; DBS: deep brain stimulation

## Discussion

Our open, retrospective study demonstrates good long-term efficacy of GPI DBS using a valid questionnaire in patients with CD over a 10-year period.

We confirmed (A) a positive effect of GPI DBS over at least a decade. Considering different disease courses, we (B) defined a subgroup of CD patients who worsened after long-term BoNT treatment and were thus candidates for GPI DBS. These were primarily patients with more complex CD-pattern and predominant torticollis. The baseline TWSTRS severity score was higher in this group than in groups investigated with regard to the long-term BoNT effect [21]. Comparing individual subitems of the TWSTRS severity score, we (C) showed significant improvement in torticollis (− 56%, p = 0.021) and displayed a favorable trend for the range of voluntary rotational head movement (− 60%, p = 0.05).

Our results are discussed in detail below:

The significant reduction in CD-severity with GPI DBS between 6.6 and 7.0 points (p < 0.001) on TWSTRS severity score is comparable to the well-known maximum effect after injection of BoNT [21, 22]. The positive effect remained stable over a 10-year period.

Recent studies reported on similar long-lasting effects of botulinum toxin up to 27 years [15].

The observed decrease in the TWSTRS severity score after implementing GPI DBS corresponds to a relative reduction of 29.1–30.8% and is in range with the BoNT treatment efficacy of larger cohorts. For example, Benecke et al. [21] showed in 463 patients with recurrent BoNT treatment an improvement in the TWSTRS severity score between 6.4 and 6.6 points (relative reduction of 38.1%) at 16-week follow-up. Comella et al. [22] demonstrated a decrease of 23% (reduction of 3.7 points in TWSTRS severity score 4 weeks after injection) in a group of 139 BoNT-treated CD patients.

The improvement after GPI DBS in the TWSTRS severity score in our study is similar to previous reports (e.g. 27.8% in Loher et al. [18]). However, some research groups found even higher improvements as described by Walsh et al. [13] (51.4%) or Cacciola et al. [16] (66.6%). In contrast to this study, the latter two showed a smaller number of patients included (n = 10) and a shorter follow-up time (37.6 months [16] and 7.8 years [13]). This might be a possible explanation for the different rates of improvement.

To our knowledge, this study is the largest CD cohort with continuous follow-up over 10 years demonstrating a positive effect of GPI DBS. In contrast, study populations of 4 [18], 8 [14, 23], and 10 [13, 15, 16] numbers are reported, with mean follow-up ranging from 30 [23] to 93.6 months [13].

The shown improvement of at least 25% is generally considered clinically significant, as described in larger previous studies of GPI DBS [11, 17]. However, the data on the efficacy of GPI DBS varies considerably. Several reasons could be given for this. On the one hand, stimulation parameters used varies widely and on the other hand patient populations are diverse with highly different observation times. For instance, the average pulse width used ranged from low (71 µs [15]) to exceptionally high (450 µs [14]) values. In our clinical experience, particularly high values are often accompanied by merely tolerated side effects such as dysarthria or ataxia. These side effects are generally attributed to a stimulation effect of the internal capsule [24]. However, currently the optimal pulse width for best stimulation effect remains unclear [25].

Now, we discuss the already mentioned eleven patients who received DBS surgery after previous long-term BoNT treatment in our clinic. Each of them had been pretreated with BoNT for at least 10 years. The relative reduction in the TWSTRS severity score one year after implementing GPI DBS was 27.4%. According to our prior determination, this decrease must be considered as clinically meaningful with a positive impact on health-related quality of life [11]. There seems to be a beneficial effect of GPI stimulation beyond BoNT in selected cases. One main reason to undergo DBS surgery could be an increasing complexity of CD over time, as described in up to 17% of patients treated with BoNT [23]. Due to the more complex presentation, probably the BoNT injections pattern became more difficult with a subsequent unsatisfactory treatment response or not tolerated side effects such as dysphagia. This assumption is supported by higher baseline TWSTRS severity scores in patients before GPI DBS compared to those who show a stable effect on BoNT. The presurgical TWSTRS severity score averaged 22.7 points, which is comparable to other studies (e.g. 21.5 points, Walsh et al. [13]). In comparison, other groups who investigated the efficacy of BoNT reported a lower mean severity score, e.g. 18 points by Benecke et al. [25].

Regarding CD-phenomenology, torticollis (90–100%), laterocollis (64–67%) and tremor (55–60%) were the most prominent clinical features. The high frequency of torticollis and tremor have been described previously [13, 30].

It is conceivable that certain predictors can be identified for patients, who might benefit from early DBS surgery.

For example, considering the different TWSTRS subitems, we found particularly a positive effect on torticollis and a statistical trend for improvement of the range of voluntary rotational head movement (p = 0.05). This appears to be important because the range of neck mobility is considered as a potential prognostic marker for GPI stimulation efficacy [19]. Torticollis is the most common presentation of cervical dystonia [16] and seems to be an important reason for patients to decide to undergo surgery. Furthermore, as exemplified in Table 2, even torticollis that initially responds well to BoNT can worsen over the years. In the analysis, a clearly significant reduction was evident with regard to torticollis in the comparison of all TWSTRS subitems. In view of the fact that torticollis is the most frequent single manifestation of CD, DBS seems to be particularly effective here.

In addition, a dystonic head tremor that cannot be sufficiently treated with BoNT injections seems to be another strong indication for DBS surgery. The majority of patients with tremor examined here showed a good treatment effect.

Finally, troublesome antecollis, which is often difficult to treat with BoNT, could be another argument in favor of GPI DBS. For example, a bilateral injection of the scaleni muscles contains a risk for dysphagia and the improvement is unsatisfactory in most cases. As shown in Table 3, there is a good response with a reduction in AC of 16.5% (and just missed significance at p = 0.051).

Relevant for patients are the number of clinical visits for sustained treatment effects. Typically, BoNT injections are given every 12 weeks, or approximately 4 times per year. In our clinic, the first postoperative visit after DBS implementation takes place 3 months after surgery and then usually semi-annually or annually. Thus, with regard to postoperative management, a reduction of clinical visits compared to BoNT of 50–75% can be expected here. However, if relevant side effects occur, the number of annual DBS visits can of course increase. The maximum number of visits was reached in a patient with stimulation-induced parkinsonoid (average 3 times per year).

## Conclusion

The strength of our work is based on the demonstration of the 10-year efficacy of GPI DBS, which also allows a comparison with the known long-term effectiveness of the primary indicated treatment form botulinum toxin. Limitations of our work include the retrospective design. Furthermore, other relevant aspects of cervical dystonia such as pain or dystonic head tremor are not considered in the TWSTRS severity score. Nevertheless, there is already a good data base that a reduced motor score in the TWSTRS can lead to a reduction in pain as well as improvement in quality of life [17].

Moreover, comparisons between DBS and BoNT will continue to be difficult, as the assessment of the effect of BoNT injection is limited by the time course (onset of the maximum effect after a few weeks) and the dependence on the treating physician.

Future long-term studies are required to better characterize the different clinical courses of CD during the various treatment modalities to define the most effective therapy for each individual patient.

In summary there is a positive long-lasting effect of GPI DBS in CD. A particularly good response to GPI DBS was observed in patients with poor primary or secondary response to BoNT. Those patients suffered mainly from more complex forms of CD and often showed predominant torticollis.

## Data Availability

The datasets used and/or analyzed during the current study are available from the corresponding author on reasonable request.

## Abbreviations

CD: Cervical dystonia
DBS: Deep brain stimulation
GPI: Globus pallidus internus
TWSTRS: Toronto Western Spasmodic Torticollis Rating Scale
BoNT: Botulinum toxin
